# Supplementary material for: Dynamic and tunable metabolite control for robust minimal-equipment assessment of serum zinc
Source: Nat Commun. 2019 Dec 4;10:5514. doi: 10.1038/s41467-019-13454-1 (PMC6892929; doi:10.1038/s41467-019-13454-1)
Supplement: Supplementary file 1 — Supplementary Information [file 41467_2019_13454_MOESM1_ESM.pdf]

Supplementary Materials for

Dynamic and tunable metabolite control for robust minimal-equipment assessment of serum zinc

Monica P. McNerney, Cirstyn L. Michel, Krishi Kishore, Janet Standeven, and Mark P. Styczynski

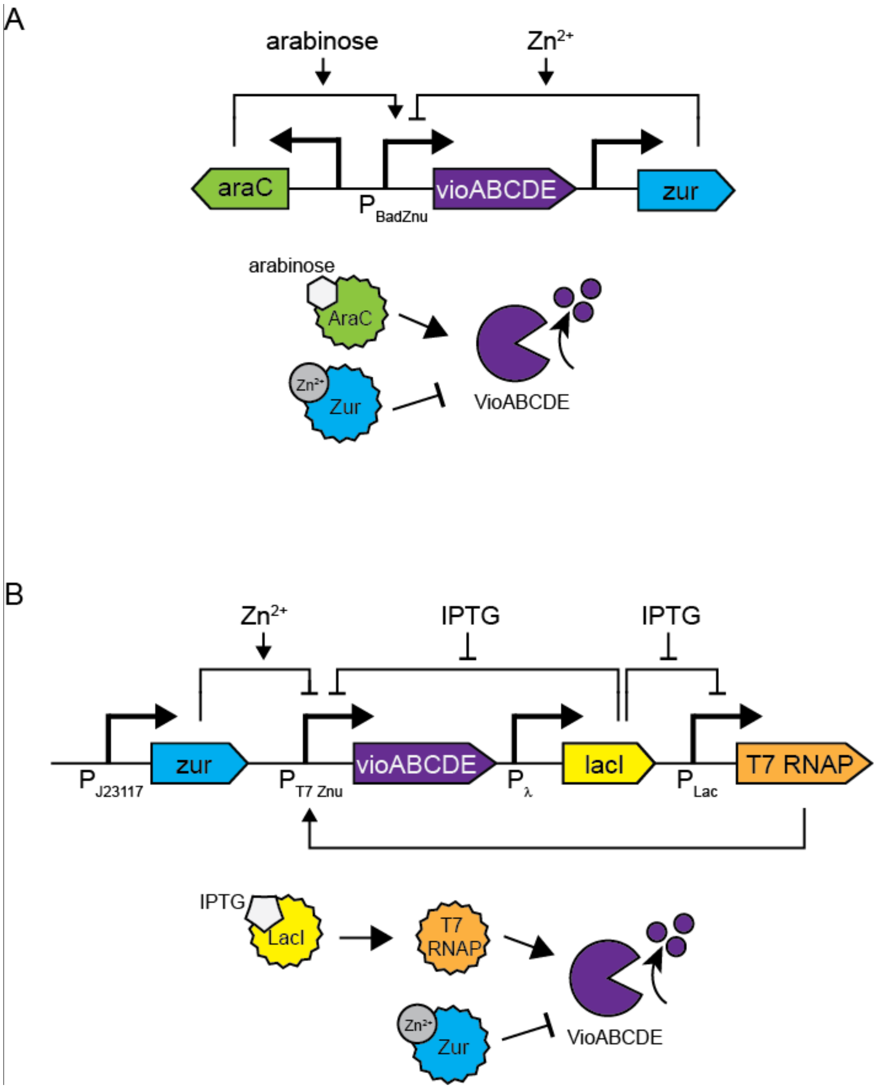

Supplementary Figure 1: AraC- and T7 RNAP-mediated violacein circuits. (A) Circuit diagram and schematic illustrating the design of a dual-input P_{Bad}-based promoter to regulate production of the violacein pathway genes. In the absence of arabinose, AraC represses pigment production. Arabinose is added as an inducer to activate AraC (switching AraC from a repressor to an activator), and Zur represses transcription when bound to zinc; thus, violacein should only be produced in low zinc conditions when arabinose is present. **(B)** Circuit diagram and schematic illustrating the design of a dual-input P_{T7}-based promoter to regulate production of the violacein pathway genes. LacI controls expression of T7 RNAP, which is required for transcription of the T7 promoter. Violacein should only be produced in low zinc conditions when IPTG is present.

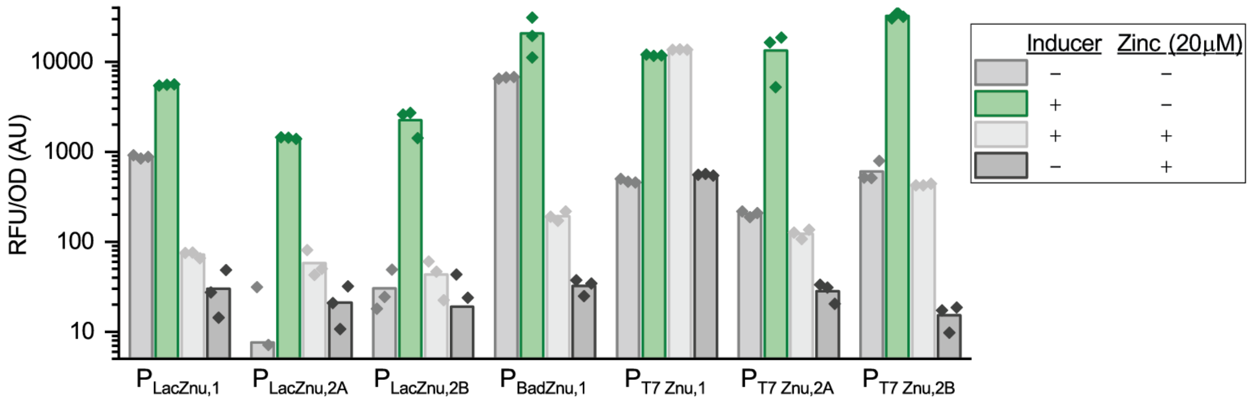

Supplementary Figure 2: Fluorescent data used for heatmap in Figure 1B. Cells containing eGFP under control of the specified promoters were grown in minimal medium containing different combinations of inducer and zinc. IPTG was used to induce both the P_{Lac}- and P_{T7}-based circuits, and arabinose was used to induce the P_{Bad}-based circuit. When indicated, zinc was added to a concentration of 20 μM. Ideally, cells should only produce eGFP in the +inducer-zinc condition. Bars represent the average of three biological replicates, which are depicted as overlaying points.

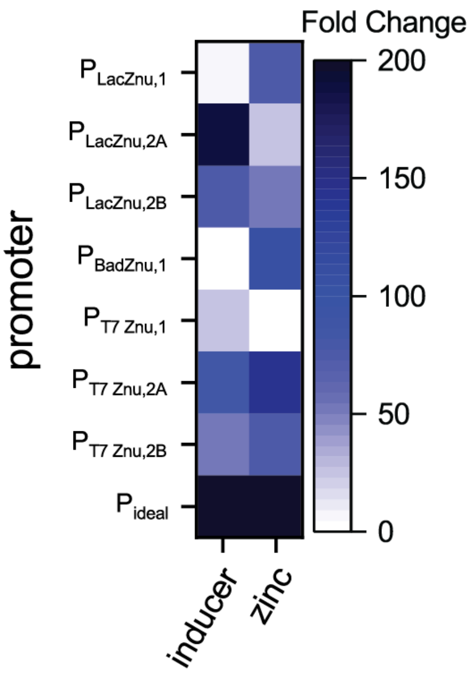

Supplementary Figure 3: Fold changes of all dually regulated promoters. Fluorescence measurements presented in Figure 1B and Supplementary Figure 2 were used to calculate fold changes of the promoters relative to both the standard inducer (either IPTG or arabinose) and to zinc. The inducer fold change was calculated as the ratio of normalized eGFP in the +inducer/-zinc state to the normalized eGFP in the -inducer/-zinc state. The zinc fold change was calculated as the ratio of normalized eGFP in the +inducer/-zinc state to the normalized eGFP in the +inducer/+zinc state.

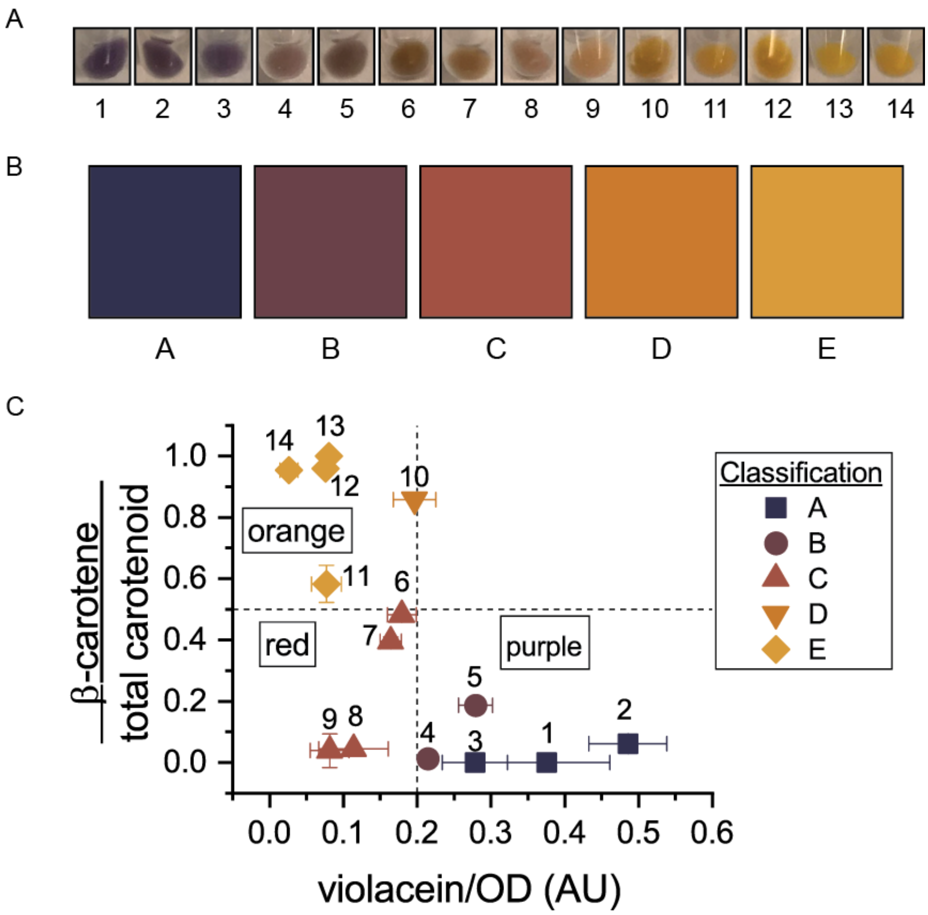

Supplementary Figure 4: Color classification of cells with different pigment concentrations. (A) Fourteen strains expressing different levels of violacein, lycopene, and β-carotene were presented to ten different people one at a time in a random order, and each person was asked to match the perceived color of each samples to different colors on a spectrum. **(B)** The color spectrum used in the survey to assess color. RGB quantification of cell pellets expressing only violacein, only lycopene, and only β-carotene was used to set the far left (A), middle (C), and far right (E) colors, respectively. The intermediate color (B) was set by averaging the RGB values of (A) and (C), and the intermediate color (D) was set by averaging the RGB values of (C) and (E). Each sample was classified as one of five colors based on the overall survey consensus. We deemed cells classified as (A) or (B) as "purple", cells classified as (C) as "red", and cells classified as (D) or (E) as "orange". **(C)** Pigment quantification of the fourteen strains and determination of quantitative color thresholds. Cell pellets contain pooled samples from biological triplicates. OD-normalized concentrations of violacein, lycopene, and β-carotene were quantified. To better enable color thresholds, we reduced carotenoid quantification into a single variable: the fraction of total carotenoid produced that is β-carotene. Error bars indicate the standard deviation. Symbol colors and shapes indicate survey results. We used these data to set quantitative color thresholds. Cells with OD-normalized violacein > 0.2 are considered "purple". Cells with OD-normalized violacein < 0.2 are considered "red" if the fraction of β-carotene is < 0.5, and "orange" if the fraction of β-carotene is > 0.5.

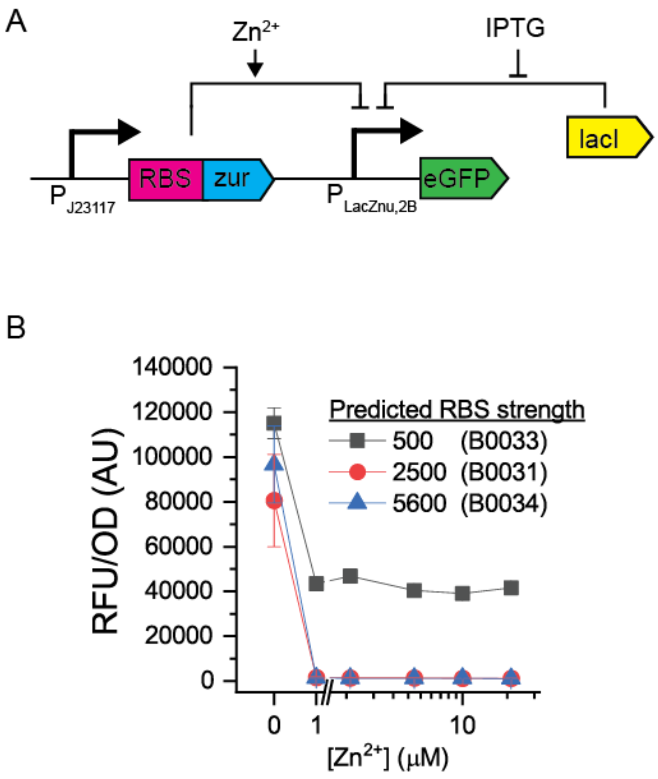

Supplementary Figure 5: Effect of Zur levels on zinc response. (A) Genetic circuit used to test the effect of RBS modulation on response to zinc. Zur is constitutively expressed from the promoter P_{J23117}, and the RBS of Zur is varied. **(B)** Fluorescent characterization showing the effect of Zur levels on expression from $P_{LacZnu,2}$. Error bars indicate standard deviation. Compared to the baseline Zur with a relative RBS strength of 2500 (arbitrary units), Zur with a decreased RBS strength of 500 leads to higher eGFP production at all zinc concentrations and minimal decrease between 1 and 20 μM zinc. Increasing the RBS strength to 5600 had no apparent effect on $P_{LacZnu,2}$ expression. RBS sequences were taken from the standard registry of parts, and their relative strengths were quantified with the RBS calculator[1].

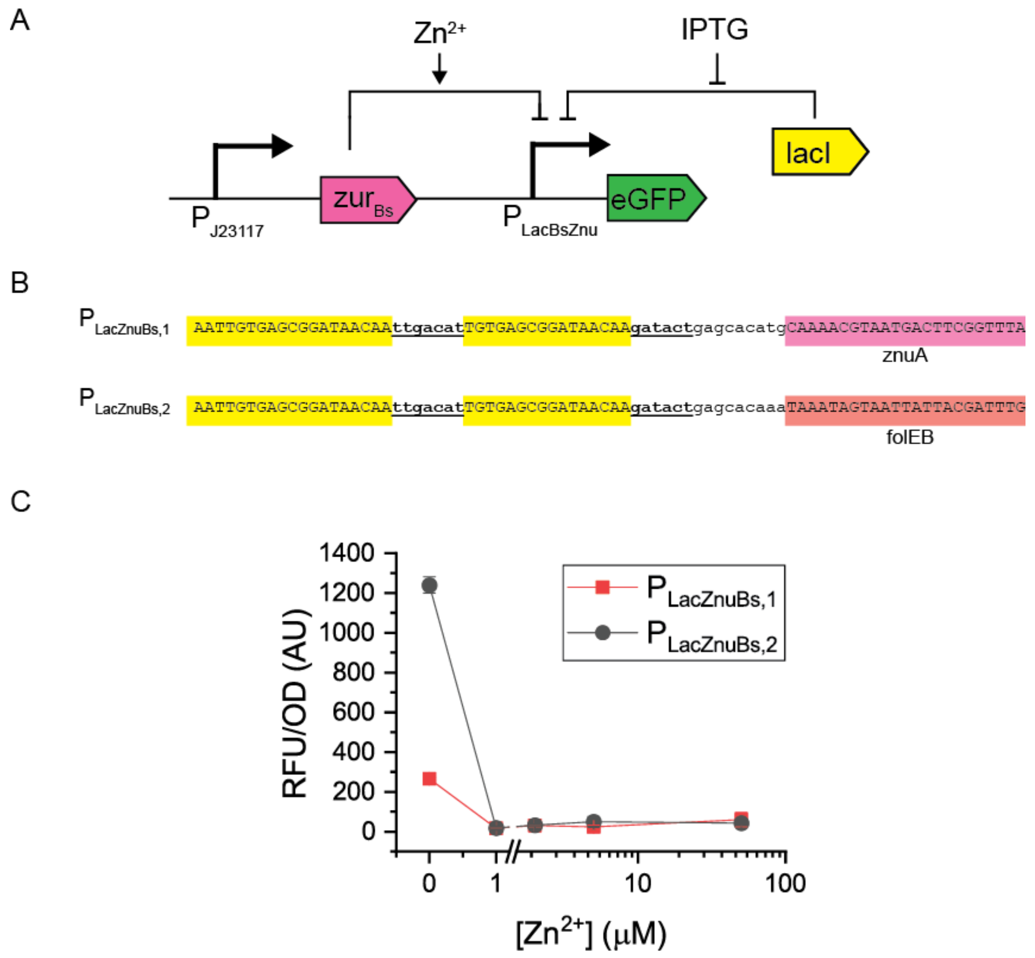

Supplementary Figure 6: Response of heterologous Zur to added zinc. (A) Genetic circuit depicting regulatory mechanism of *B. subtilis*'s Zur (Zur_{Bs}). Zur_{Bs} is constitutively expressed from the promoter P_{J23117}, and when Zur_{Bs} is bound to zinc, it binds to its cognate operator site to repress transcription. **(B)** Sequences of promoters controlled by Zur_{Bs}. Two different Zur_{Bs} operator sites, taken from either the P_{znuA} or P_{folEB} of *B. subtilus*, were cloned downstream of a Lac-inducible promoter. **(C)** Fluorescent characterization of Zur_{Bs} regulation. Error bars indicate standard deviation. Expression from both Zur_{Bs}-responsive promoters shuts off by 1 μM zinc.

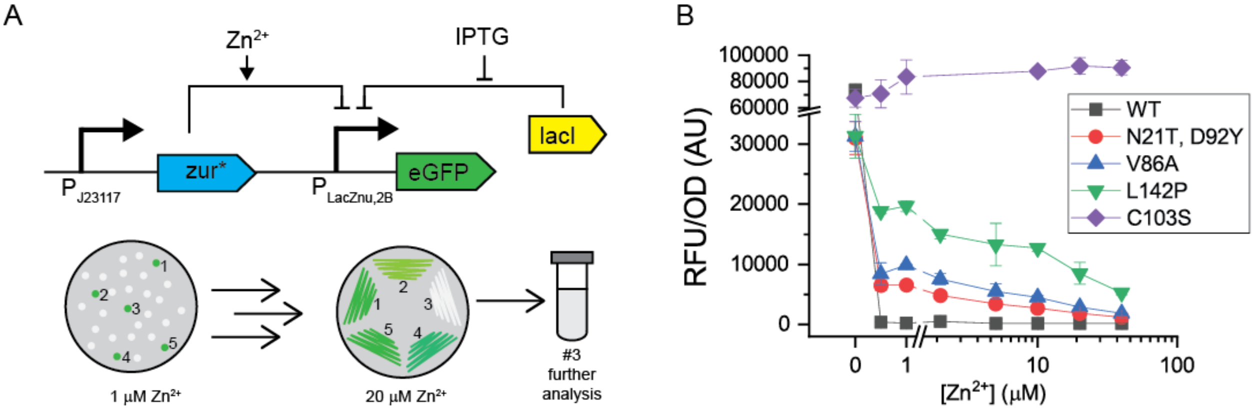

C

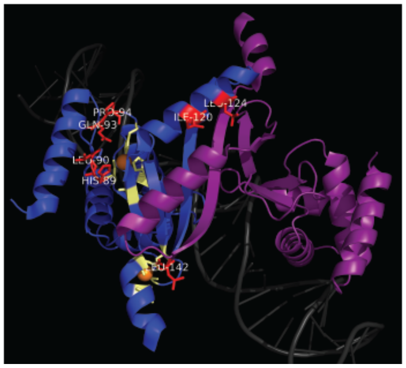

D

Residue mutated	Saturation mutagenesis result
H89	no hits
L90	no hits
Q93	no hits
P94	no hits
I120	no hits
L124	no hits
L142	1 hit (L142P)

Supplementary Figure 7: Zur mutagenesis to shift response point. (A) Plan for untargeted Zur mutagenesis and screening. Error-prone PCR was used to introduce mutations into the coding sequence of Zur, as indicated with the asterisk. Transformants were plated on M9-agar plates that contained 1 µM zinc and grown overnight. Visibly green colonies were selected and streaked onto M9-agar plates containing 20 µM zinc. Visibly white colonies were then selected for further screening. **(B)** Fluorescent characterization of mutagenesis “hits”. Error bars indicate standard deviation. Of the over 10,000 colonies screened, plasmids were isolated from the three initial “hits”, sequenced, and more thoroughly characterized. All mutants showed decreased expression between 1 and 30 µM zinc, but the fold changes were relatively small. The previously characterized mutant C103S[2] cannot bind zinc and was used to control for decreased expression caused solely by addition of zinc. **(C)** Target residues for site-directed mutagenesis of Zur. Using a crystal structure of Zur bound to DNA (PDB ID 4MTD)[2] and PyMol software[3], seven amino acid residues were selected for saturation mutagenesis, based on either their proximity to the zinc-binding domains or their role in the protein dimerization domain. For each residue, 96 colonies were inoculated into minimal media containing 1 µM zinc and grown overnight. Then, this starter culture was used to inoculate media with 20 µM zinc and grown for 12 hours. We set thresholds for “hits” as the OD-normalized eGFP fluorescence of cells grown in 1 µM zinc being both two-fold higher than cells grown in 30 µM zinc and ten-fold greater than the autofluorescence of untransformed cells. **(D)** Results of mutagenesis. Of all mutants formed, only a single colony met the requirements specified in (C). This mutant was sequenced, and the mutation (L142P) was the same mutation found in the EP-PCR mutagenesis experiments.

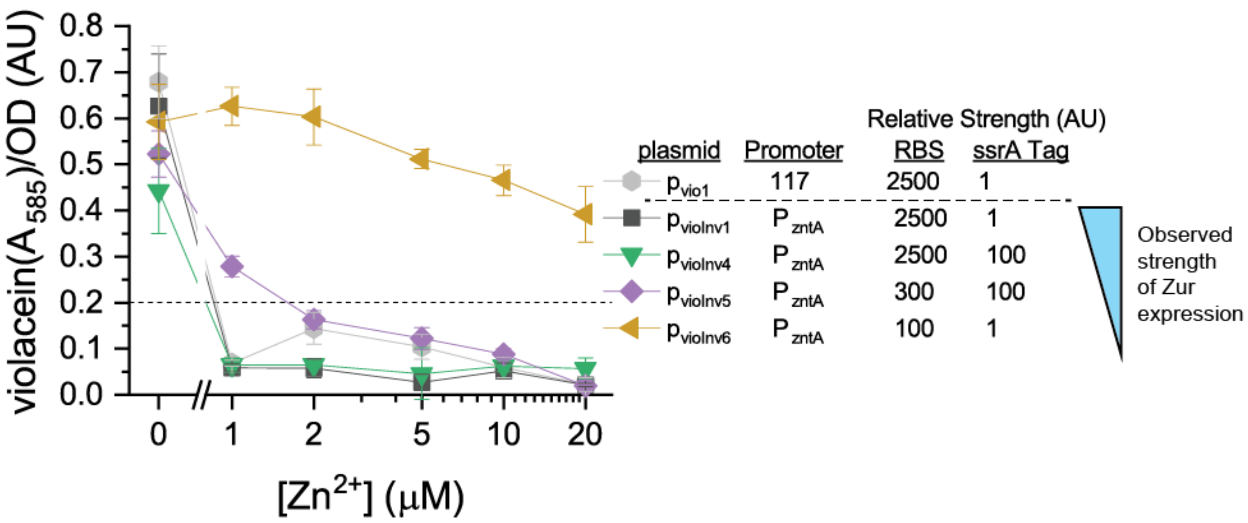

Supplementary Figure 8: Violacein quantification of inverter constructs. OD-normalized violacein for the cells depicted in Figure 3C. The dotted line indicates the threshold for visible violacein. Errors bars indicate standard deviation.

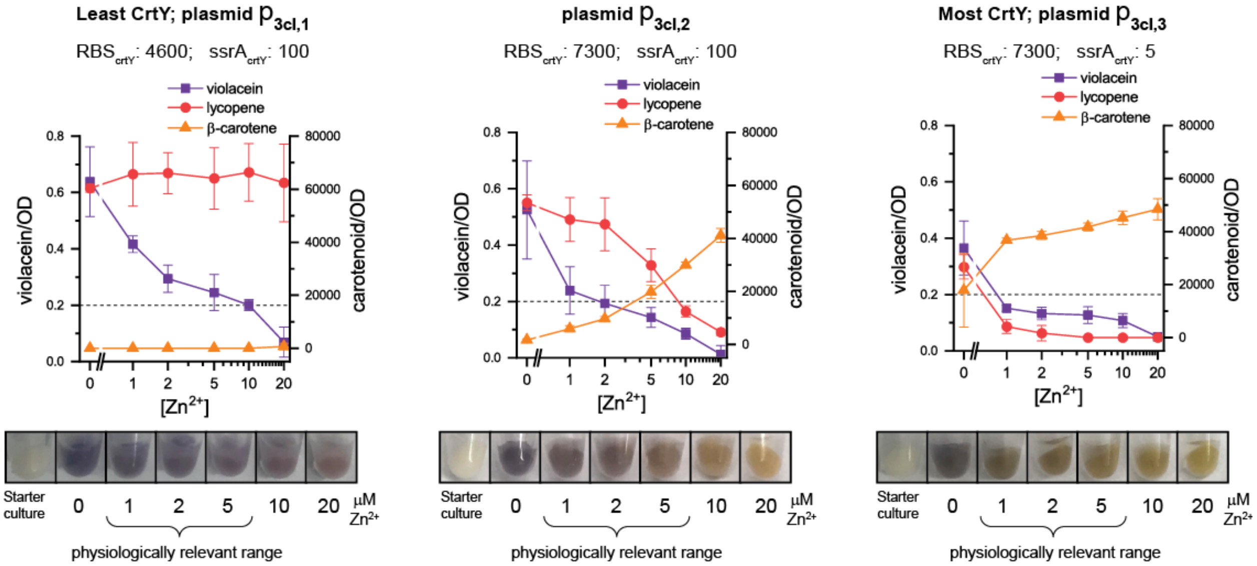

Supplementary Figure 9: Pigment quantification and visualization of three-color sensor cells grown in minimal medium containing glucose. All sensor cells have tunable color transition points, but high baseline violacein production and low overall carotenoid expression leads to murky cell coloration. The dotted line indicates the threshold for visible violacein. Error bars indicate standard deviation.

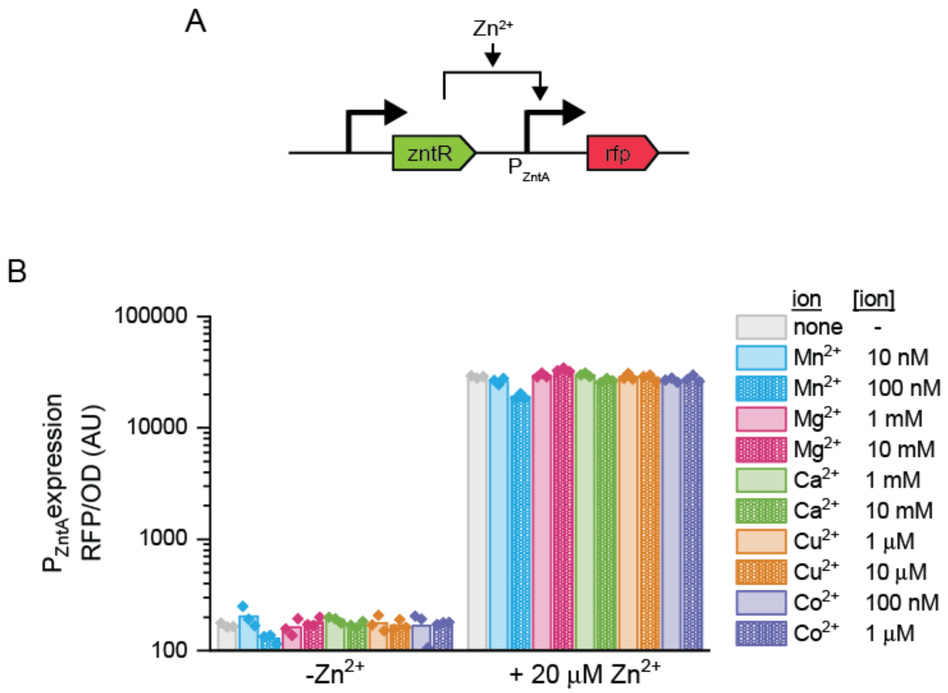

Supplementary Figure 10: Specificity of ZntR for zinc. (A) The fluorescent reporter plasmid used for assessment of ZntR/P_{ZntA} specificity. ZntR binds zinc and activates expression of P_{ZntA}, which controls RFP production. **(B)** Effect of ion addition on expression of P_{ZntA}. The fluorescent reporter plasmid in (A) was transformed into DH10B cells, and cells were inoculated into modified minimal media containing different divalent cations over a concentration range that encompasses physiologically relevant ion concentrations. In the absence of zinc, addition of other divalent cations does not induce P_{ZntA} expression. Zinc addition induces expression of P_{ZntA} in all tested conditions. Bars represent the average of three biological replicates, which are depicted as overlaying points.

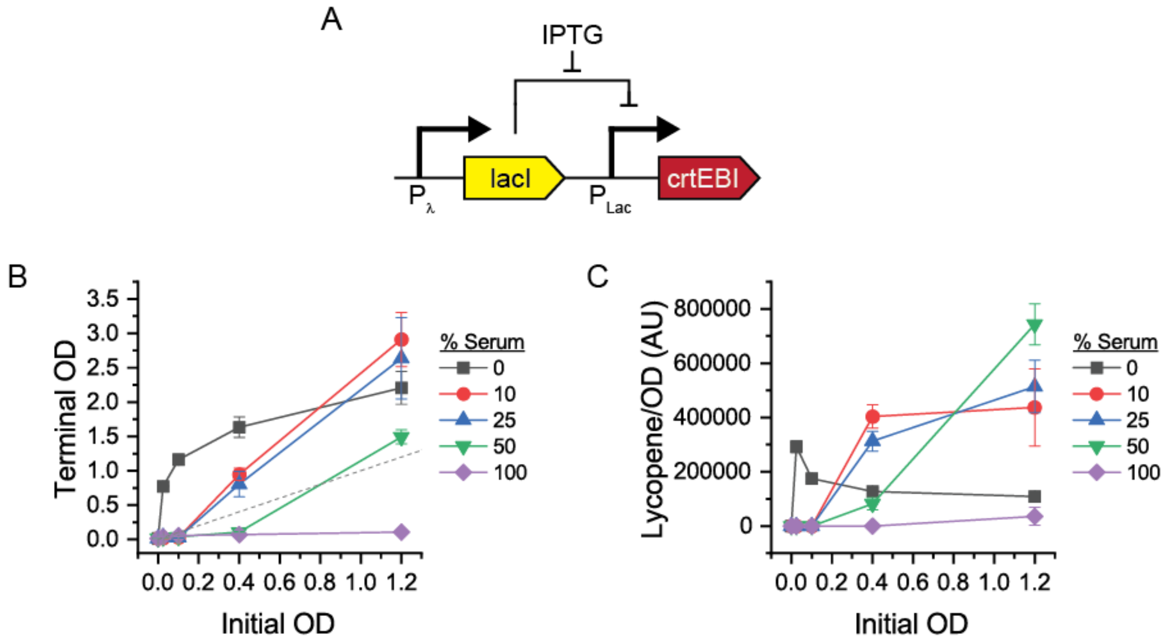

Supplementary Figure 11: Effect of serum percentage and inoculation density on cell growth and pigment production. (A) Circuit diagram of construct used to test metabolic activity. The *crtEBI* genes, which produce the red pigment lycopene, are controlled by an IPTG-inducible promoter. **(B)** Effect of serum percentage and initial inoculation density on terminal OD. For cells to grow in increasing amounts of serum, cultures must be inoculated to higher starting densities. Data points falling below the dashed gray line indicate that cells had negative changes in OD, which could indicate cell lysis. **(C)** Effect of serum percentage and initial inoculation density on terminal lycopene production. When grown in serum, if cultures are inoculated to a sufficiently high initial OD, cells produce more OD-normalized lycopene than cells grown without serum. Error bars indicate standard deviation.

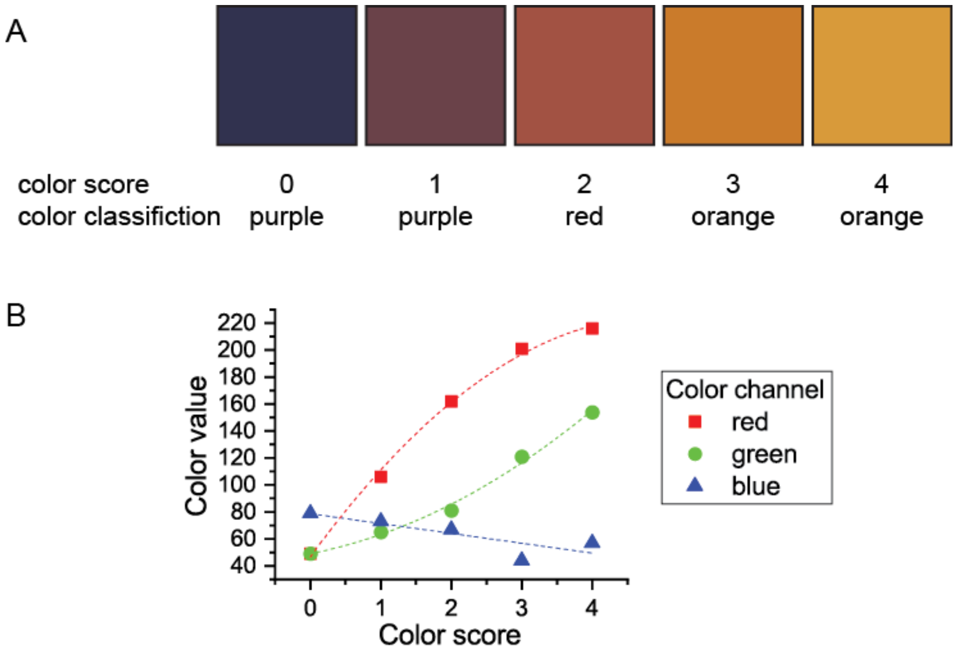

C

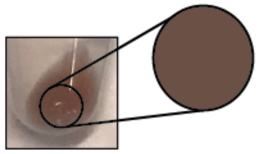

Color channel	RGB value	Color score	Average color score
red	107	0.93	1.40
blue	79	1.67	
green	69	1.59	

Supplementary Figure 12: Method for preliminary quantitative color assessment. (A) Colors and color scores that correspond with purple, red, and orange cells. Color spectrum is reproduced from Supplementary Figure 4. **(B)** Plot of the RGB values corresponding with the color bar shown in (A). From these data, we determined relationships to relate the raw color value of each channel to the color score that describes whether cells are purple, red, or orange. The x-axis represents color, with 0 and 1 corresponding with purple cells, 2 corresponding with red cells, and 3 and 4 corresponding with orange cells. **(C)** Example of photo processing pipeline. The average RGB values of each cell pellet are determined with Adobe Photoshop. For each of the red, blue, and green values, a color score is calculated using the correlations shown in (B). These three numbers are averaged to produce a single overall color score.

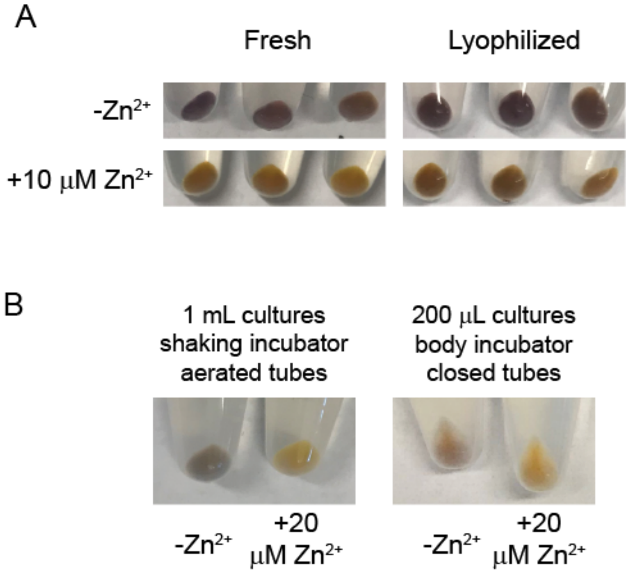

Supplementary Figure 13: Development of field-friendly test format (A) Effect of lyophilization on sensor cells without added serum. Sensor cells that were lyophilized produce color that is nearly identical to that of fresh cells. **(B)** Effect of incubation condition on lyophilized cells that were rehydrated in 25% serum containing different zinc concentrations. Cultures that are run in field-friendly conditions (small culture volumes, incubated in closed tubes with just body heat and agitation) show zinc-dependent color production similar to cultures run in standard lab conditions (1 mL culture volumes, incubated in aerated tubes in a shaking incubator).

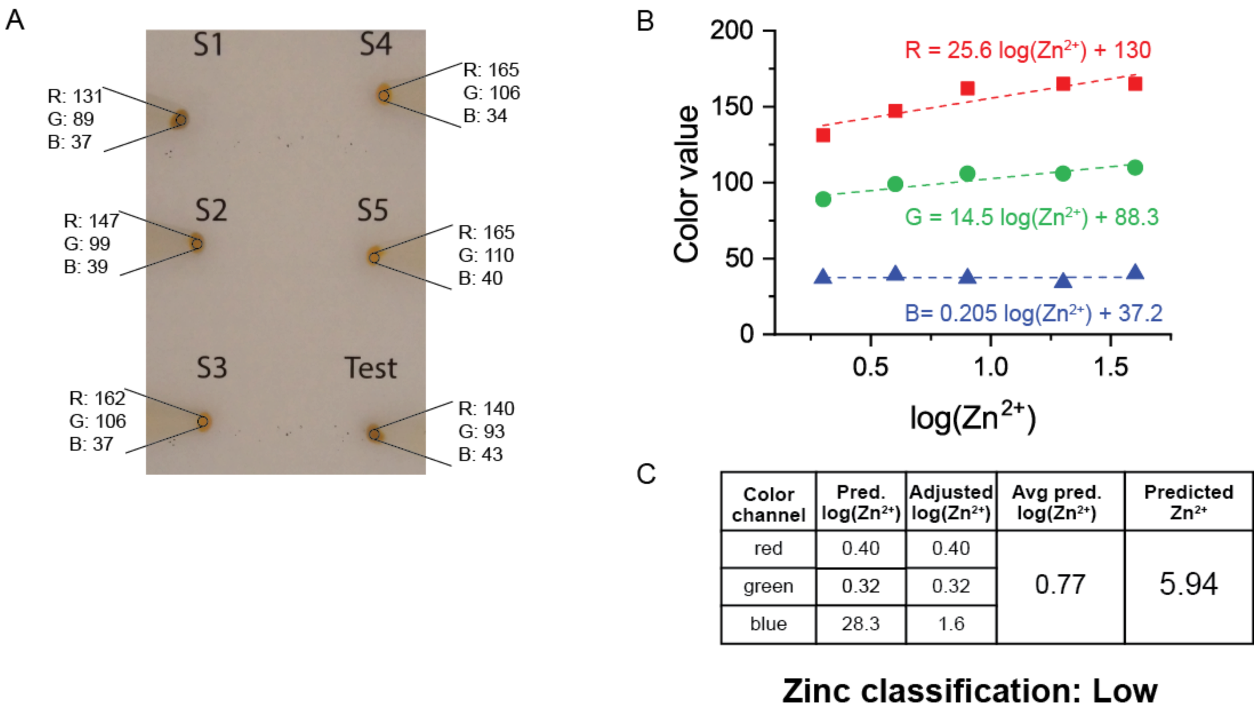

C

Color channel	Pred. $\log(Zn^{2+})$	Adjusted $\log(Zn^{2+})$	Avg pred. $\log(Zn^{2+})$	Predicted Zn^{2+}
red	0.40	0.40	0.77	5.94
green	0.32	0.32		
blue	28.3	1.6		

Zinc classification: Low

Supplementary Figure 14: Standardization approach to enable robust test assessment (A) Example picture of test that contains 5 standard reactions with known zinc concentrations and a test reaction with an unknown zinc concentration. Photoshop was used to determine the average color over a defined area in each cell pellet. **(B)** Plot of the RGB values corresponding with the colors of the standards shown in (A). From these data, we determined relationships to relate the raw color value of each channel to log of the concentration of zinc in the serum sample. **(C)** Example calculation of zinc concentration. Each correlation in (B) is used to determine the log of the concentration of zinc in the serum sample. If any of the predicted $\log(Zn^{2+})$ value falls outside of the range of the standards, it is adjusted to be equal to either the minimum or maximum standard value. The concentration of zinc is determined from the average of the calculated values. Samples are classified as either “low”, “borderline”, or “high” based on whether they fall into the range of 0 – 8 µM, 8 – 15 µM, or 15 – 40 µM, respectively.

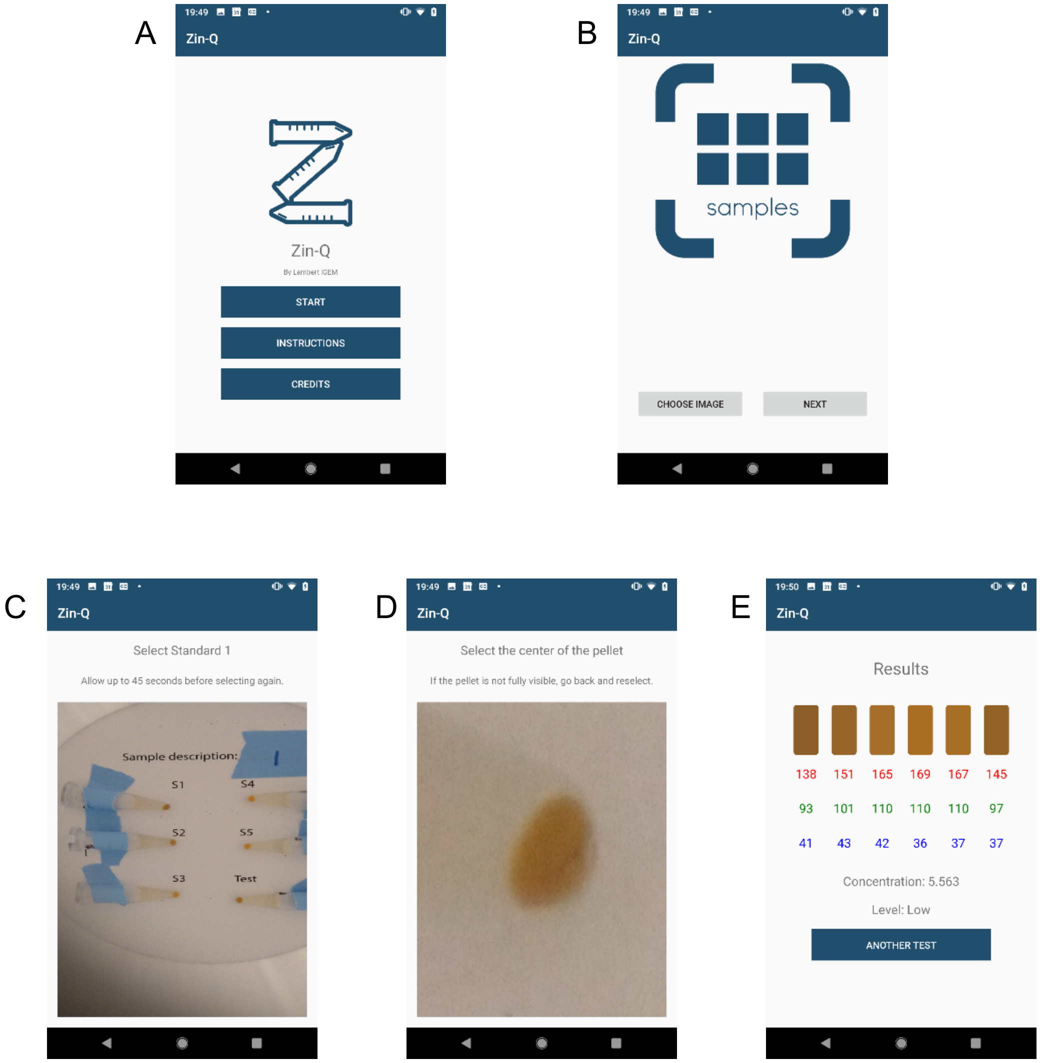

Supplementary Figure 15: Screenshots of Zin-Q smartphone app for assessing color (A) Main page of the app that contains information about the app and a button to start the quantification process. **(B)** Upon pressing "Start", the app shows a screen that has a link to "Choose Image", which allows the user to select an image from the Photo Library for analysis. **(C)** Once the user selects an image, the app prompts the user to select each of the standards and the test sample from the selected photo. **(D)** After the user taps on the specified cell pellet, a 200 x 200 pixel image taken from the region surrounding the initial tap appears, and the user is prompted to select the center of the pellet. A 20 x 20 pixel area around the user's tap is then selected, and the RGB values of these pixels are averaged and used in calculations. **(E)** After selecting all cell pellets, the app shows a results screen, which contains the RGB values used in calculations, the determined zinc concentration, and the overall zinc classification.

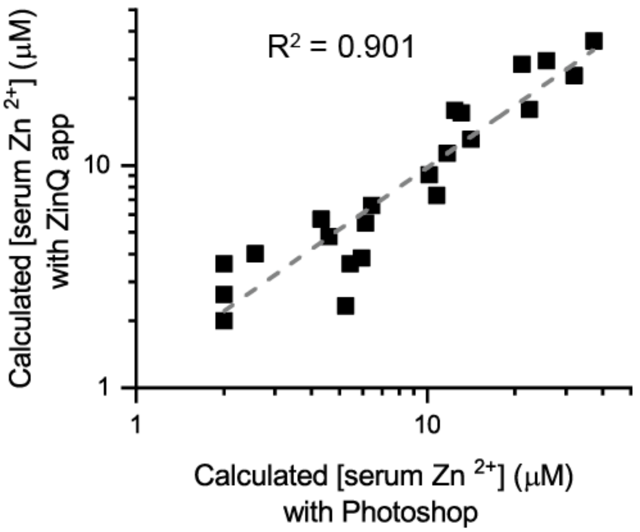

Supplementary Figure 16: Comparison of Zin-Q smartphone app to manual pellet selection with Photoshop. Results from the app are strongly correlated with assessment via manual pellet analysis via image processing software, indicating that the app can reliably quantify the color of cell pellets.

Supplementary Table 1: Sequences of all dual-input promoters used in this study. The sequences are color coded according to the schematic in Figure 1C. Lac operator sites are highlighted in yellow; the sequence of AraC- P_{Bad} promoter is highlighted in green; Zur operator sites are highlighted in blue, and the -10 σ_{70} binding site is underlined and italicized. For all P_{Lac} and P_{Bad} variants, the -10 and -35 σ_{70} binding sites are bolded and underlined. For all P_{T7} variants, the consensus T7 promoter sequence is bolded and underlined.

Promoter	Sequence
$P_{LacZnu,1}$	AATTGTGAGCGGATAACAA**TTGACAT**TGTGAGCGGATAACAA**GATACT**GAGCACAGAAGTGTGA*T*<br>*ATTAT*AACATTT
$P_{LacZnu,2A}$	AATTGTGAGCGGATAACAA**TTGACAT**TGTGAGCGGATAACAA**GATACT**GAGCACAGAAGTGTGA*T*<br>*ATTAT*AACATTTAATTGTGAGCGGATAACAA
$P_{LacZnu,2B}$	AATTGTGAGCGGATAACAA**TTGACAT**TGTGAGCGGATAACAA**GATACT**GAGCACAGAAGTGTGA*T*<br>*ATTAT*AACATTTAATTGTGAGCGGATAACAAGACTGAATTGTGAGCGCTCACAATT
$P_{BadZnu,1}$	TTATGACAACTTGACGGCTACATCATTCACTTTTTCTTCACAACCGGCACGGAACTCGCTCGGGC<br>TGGCCCCGGTGCATTTTTTAAATACCCGCGAGAAATAGAGTTGATCGTCAAAACCAACATTGCGA<br>CCGACGGTGGCGATAGGCATCCGGGTGGTGCTCAAAAGCAGCTTCGCCTGGCTGATACGTTGGTC<br>CTCGCGCCAGCTTAAGACGCTAATCCCTAACTGCTGGCGGAAAAGATGTGACAGACGCGACGGCG<br>ACAAGCAAACATGCTGTGCGACGCTGGCGATATCAAAATTGCTGTCTGCCAGGTGATCGCTGATG<br>TACTGACAAGCCTCGCGTACCCGATTATCCATCGGTGGATGGAGCGACTCGTTAATCGCTTCCAT<br>GCGCCGCAGTAACAATTGCTCAAGCAGATTTATCGCCAGCAGCTCCGAATAGCGCCCTTCCCCTT<br>GCCCGGCGTTAATGATTTGCCCAAACAGGTCGCTGAAATGCGGCTGGTGCGCTTCATCCGGGCGA<br>AAGAACCCCGTATTGGCAAATATTGACGGCCAGTTAAGCCATTCATGCCAGTAGGCGCGCGGACG<br>AAAGTAAACCCACTGGTGATACCATTCGCGAGCCTCCGGATGACGACCGTAGTGATGAATCTCTC<br>CTGGCGGGAACAGCAAAATATCACCCGGTCGGCAAACAAATTCTCGTCCCTGATTTTTCACCACC<br>CCCTGACCGCGAATGGTGAGATTGAGAATATAACCTTTCATTCCCAGCGGTCGGTCGATAAAAAA<br>ATCGAGATAACCGTTGGCCTCAATCGGCGTTAAACCCGCCACCAGATGGGCATTAAACGAGTATC<br>CCGGCAGCAGGGGATCATTTTGCGCTTCAGCCATACTTTTCATACTCCCGCCATTCAGAGAAGAA<br>ACCAATTGTCCATATTGCATCAGACATTGCCGTCACTGCGTCTTTTACTGGCTCTTCTCGCTAAC<br>CAAACCGGTAACCCCGCTTATTAAAAGCATTCTGTAACAAAGCGGGACCAAAGCCATGACAAAAA<br>CGCGTAACAAAAGTGTCTATAATCACGGCAGAAAAGTCCACATTGATTATTTGCACGGCGTCACA<br>CTTTGCTATGCCATAGCATTTTTATCCATAAGATTAGCGGATCCTAC**CTGATG**ATGAATATGAGA<br>AGTGTGA**TATTAT**AACATTT
$P_{T7Znu,1}$	**TAATACGACTCACTATAGG**AATTGTGAGCGGATAACAAGAAGTGTGA*TATTAT*AACATTT
$P_{T7Znu,2A}$	**TAATACGACTCACTATAGG**AATTGTGAGCGGATAACAAGAAGTGTGA*TATTAT*AACATTTAATTG<br>TGAGCGCTCACAATT
$P_{T7Znu,2B}$	**TAATACGACTCACTATAGG**AATTGTGAGCGGATAACAAGAAGTGTGA*TATTAT*AACATTTAATTG<br>TGAGCGGATAACAAGACTGAATTGTGAGCGCTCACAATT

Supplementary Table 2: Zinc concentration in serum isolated from individual donors. Zinc was isolated from four donors, and Chelex-100 resin was used to deplete zinc. Zinc concentration of both untreated and treated serum was measured with an ICP-MS.

	**Untreated serum**	**Chelex-100 treated serum**
**Donor 1**	13.8 μM	2.2 μM
**Donor 2**	15.6 μM	2.4 μM
**Donor 3**	15.5 μM	1.9 μM
**Donor 4**	16.8 μM	4.2 μM

Supplementary Table 3: Regulatory sequences used to control protein expression. Expression of Zur and CrtY was modulated by varying the ribosomal binding site and degradation tag of the proteins. The RBS calculator was used to assess relative RBS strength, and previously collected fluorescent data[4] was used to assess the strength of degradation tags.

**RBS controlling CrtY**		
**Predicted strength**	**Sequence**	**Origin**
4600	TCACACAGGAC	Registry of Parts: BBa_B0033
7300	TCACACAGGAAACC	Registry of Parts: BBa_B0031
**RBS controlling Zur**		
**Predicted strength**	**Sequence**	**Origin**
2500	TCACACAGGAAACC	Registry of Parts: BBa_B0031
300	GTAATATTTCGAGAATTTGAGATCA	designed for this study with RBS calculator
100	GGGACCTAATTAACCCCAAGCCAAGCGGCGT	designed for this study with RBS calculator
**Degradation tags controlling CrtY and Zur**		
**Predicted strength**	**Sequence**	**Origin**
100	GCTGCTAACGACGAAAACTACGCTCTGGCTGCTTAA	Registry of Parts: BBa_M0050
10	GCTGCTAACGACGAAAACTACAACTACGCTGACGCTTCTTAA	Registry of Parts: BBa_M0051
5	GCTGCTAACGACGAAAACTACGCTGACGCTTCTTAA	Registry of Parts: BBa_M0052

Supplementary Table 4: Regulatory proteins used to control gene expression.

Gene	Origin	Sequence
lacI	Registry of Parts: Bba_C0012; degradation tag was removed	ATGAAACCAGTAACGTTATACGATGTCGCAGAGTATGCCGGTGTCTCTTATCA<br>GACCGTTTCCCGCGTGGTGAACCAGGCCAGCCACGTTTCTGCGAAAACGCGGG<br>AAAAAGTGGAAGCGGCGATGGCGGAGCTGAATTACATTCCCAACCGCGTGGCA<br>CAACAACTGGCGGGCAAACAGTCGTTGCTGATTGGCGTTGCCACCTCCAGTCT<br>GGCCCTGCACGCGCCGTCGCAAATTGTCGCGGCGATTAAATCTCGCGCCGATC<br>AACTGGGTGCCAGCGTGGTGGTGTCGATGGTAGAACGAAGCGGCGTCGAAGCC<br>TGTAAAGCGGCGGTGCACAATCTTCTCGCGCAACGCGTCAGTGGGCTGATCAT<br>TAACTATCCGCTGGATGACCAGGATGCCATTGCTGTGGAAGCTGCCTGCACTA<br>ATGTTCCGGCGTTATTTCTTGATGTCTCTGACCAGACACCCATCAACAGTATT<br>ATTTTCTCCCATGAAGACGGTACGCGACTGGGCGTGGAGCATCTGGTCGCATT<br>GGGTCACCAGCAAATCGCGCTGTTAGCGGGCCCATTAAGTTCTGTCTCGGCGC<br>GTCTGCGTCTGGCTGGCTGGCATAAATATCTCACTCGCAATCAAATTCAGCCG<br>ATAGCGGAACGGGAAGGCGACTGGAGTGCCATGTCCGGTTTTCAACAAACCAT<br>GCAAATGCTGAATGAGGGCATCGTTCCCACTGCGATGCTGGTTGCCAACGATC<br>AGATGGCGCTGGGCGCAATGCGCGCCATTACCGAGTCCGGGCTGCGCGTTGGT<br>GCGGATATCTCGGTAGTGGGATACGACGATACCGAAGACAGCTCATGTTATAT<br>CCCGCCGTTAACCACCATCAAACAGGATTTTCGCCTGCTGGGGCAAACCAGCG<br>TGGACCGCTTGCTGCAACTCTCTCAGGGCCAGGCGGTGAAGGGCAATCAGCTG<br>TTGCCCGTCTCACTGGTGAAAAGAAAAACCACCCTGGCGCCCAATACGCAAAC<br>CGCCTCTCCCCGCGCGTTGGCCGATTCATTAATGCAGCTGGCACGACAGGTTT<br>CCCGAc
zntR	DH10B genomic DNA	ATGTATCGCATTGGTGAGCTGGCAAAAATGGCGGAAGTAACACCCGACACGAT<br>TCGTTATTACGAAAAACAGCAGATGATGGAGCATGAAGTGCGTACTGAAGGTG<br>GGTTTCGCCTATATACCGAAAGCGATCTCCAGCGATTGAAATTTATCCGCCAT<br>GCCAGACAACTAGGTTTCAGTCTGGAGTCGATCCGCGAGTTGCTGTCGATCCG<br>CATCGATCCTGAACACCATACCTGTCAGGAGTCAAAAGGCATTGTGCAGGAAA<br>GATTGCAGGAAGTCGAAGCACGGATAGCCGAGTTGCAGAGTATGCAGCGTTCC<br>TTGCAACGCCTTAACGATGCCTGTTGTGGGACTGCTCATAGCAGTGTTTATTG<br>TTCGATTCTTGAAGCTCTTGAACAAGGGGCGAGTGGCGTTAAGAGTGGTTGTT<br>GA
zur	DH10B genomic DNA	ATGGAAAAGACCACAACGCAGGAGTTATTAGCGCAGGCTGAAAAAATCTGCGC<br>GCAGCGTAATGTGCGCCTGACCCCACAGCGCCTGGAAGTGTTGCGCCTGATGA<br>GTCTCCAAGATGGCGCTATCAGCGCTTATGATCTGCTTGATTTACTGCGCGAA<br>GCTGAACCGCAAGCCAAGCCGCCAACGGTTTATCGCGCGCTGGATTTTCTGCT<br>TGAGCAAGGTTTTGTGCATAAGGTGGAATCCACCAACAGTTATGTGCTCTGTC<br>ATCTGTTCGATCAGCCCACCCATACGTCAGCCATGTTTATTTGCGATCGCTGC<br>GGCGCAGTGAAAGAAGAGTGTGCAGAAGGCGTGGAAGACATTATGCATACGCT<br>GGCGGCAAAAATGGGGTTTGCCCTGCGGCATAATGTGATTGAAGCACATGGGC<br>TCTGTGCGGCATGTGTAGAAGTGGAAGCGTGTCGTCATCCTGAACAGTGCCAG<br>CATGATCACTCTGTGCAGGTGAAAAAGAAACCGCGTTAA
**zur_{Bs}**	synthesized for this project	ATGAACGTCCAAGAAGCGCTGAACCTATTAAAAGAAAACGGATATAAATATAC<br>AAACAAACGGGAGGATATGCTcCAGCTTTTTGCTGATTCAGACAGATATCTTA<br>CCGCTAAAAACGTACTGTCTGCACTGAATGATGATTATCCAGGCTTAAGCTTT<br>GATACAATCTACAGAAATCTTTCTTTATATGAAGAACTTGGGATTTTGGAAAC<br>AACTGAGCTGTCCGGTGAAAAGCTCTTCCGATTTAAGTGTTCATTCACCCATC<br>ACCACCATCATTTTATTTGTCTTGCCTGCGGCAAAACAAAGGAAATTGAATCA<br>TGTCCGATGGACAAGCTTTGTGATGATTTGGATGGCTATCAGGTCAGCGGGCA<br>TAAATTTGAGATTTATGGCACATGCCCTGATTGTACAGCGGAAAACCAAGAAA<br>ACACTACTGCGTAA

Supplementary Table 5: Sequences of carotenoid and violacein pigment pathways.

<table>
<tr><th colspan="2">*crtEBI* operon</th></tr>
<tr><td>Origin</td><td>Genes were isolated from BBa_K274100 in the Registry of Parts.
A medium strength RBS taken from the Registry of Parts (BBa_B0032) was cloned upstream of each the *crt* genes</td></tr>
<tr><td>Annotation</td><td>Lac-inducible promoter is highlighted in green
Genes are highlighted in red, pink, and yellow (*crtE, crtB*, *crtI*, respectively).
RBS sequences are highlighted in bold</td></tr>
<tr><td colspan="2">AATTGTGAGCGGATAACAATTGACATTGTGAGCGGATAACAAGATACTGAGCACATACTAG**TCACACAGGAAAG**TA
CTAGATGACGGTCTGCGCAAAAAAACACGTTCATCTCACTCGCGATGCTGCGGAGCAGTTACTGGCTGATATTGAT
CGACGCCTTGATCAGTTATTGCCCGTGGAGGGAGAACGGATGTTGTGGGTGCCGCGATGCGTGAAGGTGCGCTGG
CACCGGGAAAACGTATTCGCCCCATGTTGCTGTTGCTGACCGCCCGCGATCTGGGTTGCGCTGTCAGCCATGACGG
ATTACTGGATTTGGCCTGTGCGGTGGAAATGGTCCACGCGGCTTCGCTGATCCTTGACGATATGCCCTGCATGGAC
GATGCGAAGCTGCGGCGCGGACGCCCTACCATTCATTCTCATTACGGAGAGCATGTGGCAATACTGGCGGCGGTTG
CCTTGCTGAGTAAAGCCTTTGGCGTAATTGCCGATGCAGATGGCCTCACGCCGCTGGCAAAAAATCGGGCGGTTTC
TGAACTGTCAAACGCCATCGGCATGCAAGGATTGGTTCAGGGTCAGTTCAAGGATCTGTCTGAAGGGGATAAGCCG
CGCAGCGCTGAAGCTATTTTGATGACGAATCACTTTAAAACCAGCACGCTGTTTTGTGCCTCCATGCAGATGGCCT
CGATTGTTGCGAATGCCTCCAGCGAAGCGCGTGATTGCCTGCATCGTTTTTCACTTGATCTTGGTCAGGCATTTCA
ACTGCTGGACGATTTGACCGATGGCATGACCGACACCGGTAAGGATAGCAATCAGGACGCCGGTAAATCGACGCTG
GTCAATCTGTTAGGCCCGAGGGCGGTTGAAGAACGTCTGAGACAACATCTTCAGCTTGCCAGTGAGCATCTCTCTG
CGGCCTGCCAACACGGGCACGCCACTCAACATTTTATTCAGGCCTGGTTTGACAAAAAACTCGCTGCCGTCAGTTA
ATAATACTAGAG**TCACACAGGAAAG**TACTAGATGAATAATCCGTCGTTACTCAATCATGCGGTCGAAACGATGGCA
GTTGGCTCGAAAAGTTTTGCGACAGCCTCAAAGTTATTTGATGCAAAAACCCGGCGCAGCGTACTGATGCTCTACG
CCTGGTGCCGCCATTGTGACGATGTTATTGACGATCAGACGCTGGGCTTTCAGGCCCGGCAGCCTGCCTTACAAAC
GCCCGAACAACGTCTGATGCAACTTGAGATGAAAACGCGCCAGGCCTATGCAGGATCGCAGATGCACGAACCGGCG
TTTGCGGCTTTTCAGGAAGTGGCTATGGCTCATGATATCGCCCCGGCTTACGCGTTTGATCATCTGGAAGGCTTCG
CCATGGATGTACGCGAAGCGCAATACAGCCAACTGGATGATACGCTGCGCTATTGCTATCACGTTGCAGGCGTTGT
CGGCTTGATGATGGCGCAAATCATGGGCGTGCGGGATAACGCCACGCTGGACCGCGCCTGTGACCTTGGGCTGGCA
TTTCAGTTGACCAATATTGCTCGCGATATTGTGGACGATGCGCATGCGGGCCGCTGTTATCTGCCGGCAAGCTGGC
TGGAGCATGAAGGTCTGAACAAAGAGAATTATGCGGCACCTGAAAACCGTCAGGCGCTGAGCCGTATCGCCCGTCG
TTTGGTGCAGGAAGCAGAACCTTACTATTTGTCTGCCACAGCCGGCCTGGCAGGGTTGCCCCTGCGTTCCGCCTGG
GCAATCGCTACGGCGAAGCAGGTTTACCGGAAAATAGGTGTCAAAGTTGAACAGGCCGGTCAGCAAGCCTGGGATC
AGCGGCAGTCAACGACCACGCCCGAAAAATTAACGCTGCTGCTGGCCGCCTCTGGTCAGGCCCTTACTTCCCGGAT
GCGGGCTCATCCTCCCCGCCCTGCGCATCTCTGGCAGCGCCCGCTCTAATAATACTAGAG**TCACACAGGAAAG**TAC
TAGATGAAACCAACTACGGTAATTGGTGCAGGCTTCGGTGGCCTGGCACTGGCAATTCGTCTACAAGCTGCGGGA
TCCCCGTCTTACTGCTTGAACAACGTGATAAACCCGGCGGTCGGGCTTATGTCTACGAGGATCAGGGGTTTACCTT
TGATGCAGGCCCGACGGTTATCACCGATCCCAGTGCCATTGAAGAACTGTTTGCACTGGCAGGAAAACAGTTAAAA
GAGTATGTCGAACTGCTGCCGGTTACGCCGTTTTACCGCCTGTGTTGGGAGTCAGGGAAGGTCTTTAATTACGATA
ACGATCAAACCCGGCTCGAAGCGCAGATTCAGCAGTTTAATCCCCGCGATGTCGAAGGTTATCGTCAGTTTCTGGA
CTATTCACGCGCGGTGTTTAAAGAAGGCTATCTAAAGCTCGGTACTGTCCCTTTTTTATCGTTCAGAGACATGCTT
CGCGCCGCACCTCAACTGGCGAAACTGCAAGCATGGAGAAGCGTTTACAGTAAGGTTGCCAGTTACATCGAAGATG
AACATCTGCGCCAGGCGTTTTCTTTCCACTCGCTGTTGGTGGGCGGCAATCCCTTCGCCACCTCATCCATTTATAC
GTTGATACACGCGCTGGAGCGTGAGTGGGGCGTCTGGTTTCCGCGTGGCGGCACCGGCGCATTAGTTCAGGGGATG
ATAAAGCTGTTTCAGGATCTGGGTGGCGAAGTCGTGTTAAACGCCAGAGTCAGCCATATGGAAACGACAGGAAACA
AGATTGAAGCCGTGCATTTAGAGGACGGTCGCAGGTTCCTGACGCAAGCCGTCGCGTCAAATGCAGATGTGGTTCA
TACCTATCGCGACCTGTTAAGCCAGCACCCTGCCGCGGTTAAGCAGTCCAACAAACTGCAAACTAAGCGCATGAGT
AACTCTCTGTTTGTGCTCTATTTTGGTTTGAATCACCATCATGATCAGCTCGCGCATCACACGGTTTGTTTCGGCC
CGCGTTACCGCGAGCTGATTGACGAAATTTTTAATCATGATGGCCTCGCAGAGGACTTCTCACTTTATCTGCACGC
GCCCTGTGTCACGGATTCGTCACTGGCGCCTGAAGGTTGCGGCAGTTACTATGTGTTGGCGCCGGTGCCGCATTTA
GGCACCGCGAACCTCGACTGGACGGTTGAGGGGCCAAAACTACGCGACCGTATTTTTGCGTACCTTGAGCAGCATT
ACATGCCTGGCTTACGGAGTCAGCTGGTCACGCACCGGATGTTTACGCCGTTTGATTTTCGCGACCAGCTTAATGC</td></tr>
</table>

<table>
<tr><td colspan="2">CTATCATGGCTCAGCCTTTTCTGTGGAGCCCGTTCTTACCCAGAGCGCCTGGTTTCGGCCGCATAACCGCGATAAA
ACCATTACTAATCTCTACCTGGTCGGCGCAGGCACGCATCCCGGCGCAGGCATTCCTGGCGTCATCGGCTCGGCAA
AAGCGACAGCAGGTTTGATGCTGGAGGATCTGATATAATAA</td></tr>
<tr><td colspan="2">***vioABCDE* expression**</td></tr>
<tr><td>Origin</td><td>Registry of Parts: BBa_K274002</td></tr>
<tr><td>Annotation</td><td>Genes are highlighted in red, pink, yellow, green, and blue (*vioA*, *vioB*, *vioC*, *vioD*, and *vioE* respectively).
RBS sequences are highlighted in bold</td></tr>
<tr><td colspan="2">**TTAAGGAGGTAAAAAAA**ATGAAACATTCTTCCGATATCTGCATTGTTGGTGCTGGTATTTCTGGTTTGACGTGCGC
AAGCCATCTGCTGGACAGCCCGGCATGCCGTGGTCTGAGCCTGCGTATCTTTGACATGCAGCAAGAAGCCGGTGGC
CGTATCCGCAGCAAAATGCTGGATGGTAAGGCAAGCATTGAACTGGGCGCAGGTCGCTACTCCCCTCAGTTGCACC
CGCATTTCCAAAGCGCAATGCAGCACTATAGCCAAAAGAGCGAAGTCTATCCGTTCACCCAGTTGAAGTTCAAATC
TCACGTGCAGCAAAAGCTGAAGCGCGCCATGAATGAACTGTCCCCGCGTCTGAAAGAGCATGGTAAAGAGAGCTTT
TTGCAGTTTGTCAGCCGTTATCAAGGTCACGATAGCGCGGTTGGTATGATCCGCTCTATGGGTTACGACGCACTGT
TCCTGCCGGATATCAGCGCAGAAATGGCCTACGACATTGTGGGTAAGCACCCGGAGATCCAGAGCGTGACGGACAA
CGACGCGAACCAATGGTTTGCAGCGGAAACGGGCTTTGCTGGTCTGATTCAGGGCATCAAGGCTAAGGTTAAGGCG
GCAGGTGCGCGTTTTAGCCTGGGTTATCGTCTGCTGAGCGTCCGTACCGACGGTGACGGCTACCTGCTGCAACTGG
CAGGTGACGACGGCTGGAAACTGGAGCACCGTACCCGCCATCTGATTCTGGCGATTCCGCCGAGCGCGATGGCGGG
TTTGAATGTTGATTTTCCAGAAGCCTGGTCCGGTGCGCGCTATGGCAGCCTGCCGCTGTTTAAGGGCTTTCTGACG
TACGGTGAGCCGTGGTGGTTGGACTACAAACTGGACGATCAGGTGCTGATTGTTGACAACCCGCTGCGCAAAATCT
ATTTCAAAGGCGATAAGTACCTGTTCTTCTATACCGATAGCGAGATGGCGAATTACTGGCGCGGTTGTGTCGCGGA
GGGCGAGGACGGTTACCTGGAGCAAATTCGCACCCATTTGGCTAGCGCACTGGGTATCGTCCGTGAACGTATCCCG
CAACCGCTGGCACACGTTCACAAGTATTGGGCGCACGGCGTTGAGTTTTGCCGTGATTCTGATATTGACCACCCGA
GCGCACTGTCTCATCGCGACAGCGGTATCATCGCGTGCTCCGATGCGTACACGGAGCATTGTGGTTGGATGGAGGG
CGGTCTGCTGAGCGCCCGTGAGGCAAGCCGTCTGCTGTTGCAGCGTATCGCCGCGTGA**TTAAGGAGGTAAAAAAA**A
TGAGCATTCTGGATTTCCCGCGTATCCACTTCCGTGGCTGGGCCCGTGTCAATGCGCCGACCGCGAACCGCGATCC
GCACGGCCACATCGATATGGCCAGCAATACCGTGGCGATGGCGGGTGAGCCGTTCGACCTGGCACGCCATCCTACG
GAGTTCCACCGTCACCTGCGCTCCCTGGGTCCGCGCTTCGGCTTGGATGGTCGTGCTGACCCGGAAGGCCCGTTCA
GCCTGGCCGAGGGCTACAACGCTGCCGGTAACAACCACTTTTCGTGGGAGAGCGCAACCGTTAGCCACGTGCAATG
GGATGGCGGTGAGGCGGATCGTGGTGACGGTCTGGTCGGTGCTCGTTTGGCACTGTGGGGTCACTACAATGATTAT
CTGCGTACCACCTTCAATCGTGCTCGTTGGGTCGACAGCGACCCGACGCGCCGTGACGCTGCACAAATCTATGCGG
GCCAATTCACCATTAGCCCGGCTGGTGCCGGTCCGGGTACGCCGTGGCTGTTTACGGCAGACATTGATGATAGCCA
TGGTGCACGTTGGACGCGTGGCGGCCACATTGCAGAGCGTGGCGGCCACTTCTTGGATGAAGAGTTTGGTCTGGCA
CGCCTGTTTCAGTTCTCTGTGCCGAAAGATCACCCACATTTTCTGTTTCACCCGGGTCCGTTTGATTCCGAGGCCT
GGCGTCGTCTGCAATTGGCTCTGGAGGATGACGACGTTCTGGGTCTGACCGTGCAATATGCGTTGTTCAATATGAG
CACCCCGCCTCAGCCGAACAGCCCGGTTTTTCACGATATGGTCGGTGTTGTCGGTCTGTGGCGTCGTGGTGAACTG
GCGAGCTACCCGGCTGGTCGTCTGCTGCGTCCGCGTCAACCGGGTCTGGGTGACCTGACCCTGCGCGTCAACGGTG
GTCGCGTTGCGCTGAATTTGGCGTGTGCCATTCCGTTCAGCACTCGTGCCGCGCAGCCAAGCGCACCGGACCGCCT
GACCCCGGACCTGGGTGCCAAACTGCCGCTGGGCGATCTGCTGCTGCGTGATGAGGACGGCGCACTGTTGGCACGT
GTGCCGCAGGCTCTGTACCAAGACTATTGGACGAATCACGGTATTGTGGACCTGCCGCTGCTGCGCGAACCGCGTG
GTAGCTTGACCCTGAGCAGCGAACTGGCGGAGTGGCGTGAGCAAGACTGGGTCACCCAAAGCGACGCGTCTAACCT
GTACCTGGAGGCACCGGATCGCCGTCACGGTCGCTTTTTCCCTGAGAGCATCGCGCTGCGCAGCTACTTTCGCGGT
GAAGCGCGTGCGCGTCCGGATATCCCGCATCGTATCGAGGGCATGGGCCTGGTCGGCGTCGAATCTCGTCAGGATG
GCGACGCTGCGGAATGGCGTCTGACGGGTCTGCGTCCGGGTCCGGCACGCATTGTTCTGGACGATGGTGCCGAGGC
GATCCCTCTGCGTGTTCTGCCTGACGATTGGGCGCTGGATGACGCGACCGTCGAAGAAGTGGATTACGCCTTTTTG
TACCGCCACGTTATGGCGTATTACGAGCTGGTGTATCCATTCATGAGCGACAAGGTGTTTTCCCTGGCTGATCGTT
GCAAATGTGAAACGTACGCACGTCTGATGTGGCAGATGTGTGATCCGCAGAACCGCAACAAGTCCTATTACATGCC
GAGCACCCGCGAACTGTCGGCACCGAAAGCTCGTTTGTTCTTGAAGTATCTGGCCCACGTGGAAGGCCAGGCACGC
CTGCAAGCACCTCCGCCAGCGGGTCCGGCACGCATTGAATCTAAAGCCCAGTTGGCGGCAGAGCTGCGTAAAGCCG
TCGACCTGGAGCTGTCTGTGATGCTGCAATACCTGTACGCGGCGTATAGCATTCCGAACTATGCACAGGGCCAACA
ACGTGTTCGTGACGGTGCGTGGACCGCCGAGCAGCTGCAACTGGCGTGCGGTAGCGGTGACCGTCGCCGTGATGGC
GGTATTCGTGCAGCACTGCTGGAAATTGCTCATGAAGAAATGATTCATTACCTGGTCGTTAACAACCTGCTGATGG
CCCTGGGCGAGCCGTTCTACGCGGGTGTCCCGCTGATGGGCGAAGCGGCACGTCAGGCGTTTGGCCTGGACACCGA
GTTCGCTCTGGAACCGTTTAGCGAAAGCACGCTGGCACGTTTTGTTCGTCTGGAATGGCCGCACTTTATCCCAGCA
CCGGGCAAATCCATCGCGGACTGCTATGCCGCCATTCGTCAGGCGTTTTTGGATCTGCCGGACTTGTTTGGTGGCG</td></tr>
</table>

AGGCAGGTAAGCGTGGCGGTGAACACCACCTGTTCCTGAATGAGCTGACCAACCGTGCGCATCCGGGTTATCAACT
GGAAGTTTTCGATCGCGACTCGGCGCTGTTTGGTATTGCATTTGTGACCGATCAGGGCGAAGGTGGCGCTCTGGAC
AGCCCGCACTACGAACATAGCCATTTTCAACGTCTGCGTGAAATGAGCGCGCGTATCATGGCTCAAAGCGCACCGT
TCGAACCGGCGCTGCCGGCGTTGCGTAATCCGGTTCTGGATGAGAGCCCGGGTTGCCAACGTGTCGCAGACGGTCG
TGCGCGTGCGCTGATGGCATTGTACCAAGGCGTTTATGAGCTGATGTTTGCGATGATGGCGCAGCACTTCGCCGTG
AAACCGCTGGGTAGCTTGCGTCGCAGCCGCCTGATGAACGCAGCAATCGATCTGATGACCGGTCTGTTGCGTCCGC
TGAGCTGCGCGCTGATGAACCTGCCAAGCGGCATCGCCGGTCGCACGGCCGGTCCGCCGCTGCCGGGTCCGGTTGA
CACCCGTAGCTATGACGACTACGCGCTGGGCTGTCGCATGCTGGCACGCCGTTGCGAGCGTCTGCTGGAGCAGGCG
AGCATGCTGGAACCGGGTTGGCTGCCGGATGCGCAGATGGAGCTGCTGGATTTCTATCGTCGCCAAATGCTGGACT
TGGCGTGCGGCAAACTGAGCCGCGAGGCCTAAGGATCC**TTAAGGAGGTAAAAAAA**ATGAAACGTGCGATTATCGTT
GGTGGCGGCCTGGCGGGTGGCCTGACCGCGATCTACCTGGCGAAGCGTGGCTACGAAGTGCACGTCGTGGAGAAGC
GTGGTGATCCTCTGCGCGATCTGAGCTCTTACGTGGACGTTGTTAGCAGCCGTGCGATCGGCGTGAGCATGACCGT
TCGTGGTATCAAGAGCGTTTTGGCTGCGGGCATTCCGCGTGCAGAGCTGGATGCGTGTGGCGAACCGATCGTGGCA
ATGGCTTTCTCCGTGGGTGGTCAGTATCGCATGCGCGAACTGAAGCCGTTGGAGGATTTCCGTCCGCTGAGCTTGA
ACCGTGCGGCGTTTCAAAAGCTGCTGAACAAATACGCGAACCTGGCAGGCGTTCGTTACTACTTTGAGCATAAGTG
CCTGGATGTTGACCTGGATGGTAAGAGCGTGTTGATTCAGGGCAAAGATGGTCAGCCGCAGCGTCTGCAAGGTGAC
ATGATTATCGGTGCGGATGGCGCCCACAGCGCCGTCCGTCAGGCGATGCAGAGCGGCCTGCGTCGTTTCGAGTTCC
AGCAAACGTTCTTCCGCCATGGCTACAAAACCCTGGTTTTGCCGGACGCGCAAGCACTGGGTTACCGTAAAGACAC
GCTGTACTTTTTCGGCATGGATTCCGGTGGCCTGTTCGCGGGTCGTGCGGCTACGATCCCAGATGGTAGCGTCAGC
ATCGCCGTTTGCCTGCCGTACTCGGGTAGCCCTTCCCTGACGACCACCGACGAACCGACGATGCGTGCGTTCTTCG
ATCGTTACTTCGGTGGCCTGCCGCGTGACGCGCGTGACGAAATGCTGCGTCAGTTTCTGGCGAAGCCGAGCAACGA
CCTGATTAACGTGCGCTCTAGCACCTTTCACTATAAGGGTAATGTGCTGTTGCTGGGTGATGCTGCGCATGCGACT
GCGCCGTTCCTGGGTCAGGGTATGAACATGGCGCTGGAGGACGCCCGCACGTTTGTCGAGCTGCTGGACCGCCACC
AGGGCGACCAAGACAAAGCCTTTCCGGAGTTCACGGAGCTGCGCAAAGTCCAGGCAGACGCAATGCAAGACATGGC
TCGCGCCAACTATGACGTTTTGAGCTGCTCGAACCCGATCTTTTTCATGCGTGCGCGTTACACGCGTTACATGCAT
TCCAAGTTTCCGGGCCTGTATCCGCCGGATATGGCCGAGAAACTGTACTTTACGAGCGAGCCGTACGATCGTCTGC
AACAAATCCAGCGTAAACAGAATGTTTGGTACAAGATTGGTCGCGTGAATTGAAGATCT**TTAAGGAGGTAAAAAAA**
ATGAAGATTCTGGTCATTGGTGCTGGTCCAGCTGGTCTGGTTTTCGCATCCCAACTGAAGCAGGCACGCCCTTTGT
GGGCCATTGACATCGTGGAGAAGAATGACGAGCAAGAAGTGCTGGGCTGGGGTGTCGTGCTGCCTGGCCGTCCGGG
TCAGCACCCGGCGAACCCGCTGTCCTATCTGGATGCACCGGAGCGTCTGAATCCGCAATTTCTGGAGGACTTCAAA
CTGGTGCATCATAATGAGCCGTCCTTGATGTCCACGGGCGTTTTGTTGTGCGGCGTGGAGCGTCGCGGTCTGGTTC
ACGCGCTGCGCGATAAGTGCCGCAGCCAAGGCATTGCTATTCGTTTCGAAAGCCCGTTGCTGGAACACGGTGAGCT
GCCGCTGGCGGACTATGATCTGGTGGTCCTGGCTAATGGTGTTAATCACAAAACCGCGCATTTCACCGAGGCTCTG
GTCCCGCAGGTGGACTACGGCCGCAATAAGTACATTTGGTATGGCACTAGCCAGCTGTTCGATCAGATGAATCTGG
TTTTTCGTACCCATGGTAAAGATATCTTTATCGCGCATGCCTATAAGTATAGCGATACCATGAGCACGTTCATTGT
CGAATGTAGCGAAGAGACTTACGCACGCGCACGCCTGGGCGAAATGTCCGAAGAGGCGAGCGCAGAATACGTTGCT
AAGGTGTTCCAGGCCGAGCTGGGTGGTCACGGCCTGGTGAGCCAGCCGGGTCTGGGTTGGCGTAACTTCATGACGT
TGTCTCATGACCGTTGTCATGATGGTAAGTTGGTTCTGCTGGGTGACGCGCTGCAAAGCGGTCACTTTAGCATCGG
CCACGGCACCACGATGGCCGTGGTGGTGGCGCAGCTGCTGGTTAAAGCGCTGTGTACCGAAGATGGTGTGCCTGCC
GCGCTGAAACGTTTCGAAGAGCGTGCCCTGCCGCTGGTGCAGTTGTTCCGTGGCCACGCAGACAACAGCCGCGTTT
GGTTCGAAACCGTCGAAGAGCGCATGCACCTGTCCTCGGCGGAATTTGTGCAAAGCTTCGACGCACGCCGCAAAAG
CCTGCCGCCGATGCCGGAAGCACTGGCGCAGAATCTGCGTTATGCTTTGCAGCGCTGATGATCA**TTAAGGAGGTAA**
AAAAAATGGAGAACCGTGAGCCACCACTGTTGCCAGCCCGTTGGAGCAGCGCCTATGTCTCTTATTGGAGCCCGAT
GCTGCCGGATGACCAGCTGACCAGCGGCTATTGCTGGTTCGACTATGAACGTGACATCTGTCGTATTGACGGCCTG
TTCAATCCGTGGAGCGAGCGTGATACTGGTTATCGCCTGTGGATGTCGGAGGTTGGTAATGCGGCCAGCGGCCGTA
CCTGGAAACAAAAAGTCGCCTATGGTCGTGAGCGTACCGCCCTGGGTGAACAGCTGTGTGAGCGTCCGCTGGATGA
TGAGACTGGCCCTTTTGCCGAATTGTTCCTGCCACGCGATGTCCTGCGCCGTCTGGGTGCCCGTCACATTGGCCGT
CGCGTGGTTCTGGGTCGCGAAGCGGACGGTTGGCGTTACCAGCGCCCAGGTAAAGGTCCGAGCACCCTGTACCTGG
ATGCGGCGAGCGGCACTCCACTGCGCATGGTCACCGGCGATGAAGCGTCGCGTGCAAGCCTGCGTGATTTTCCGAA
TGTGAGCGAGGCGGAGATCCCGGACGCGGTTTTCGCGGCCAAGCGCTAA

***crtY* gene**

Origin	Registry of Parts: BBa_K118013

ATGCAACCGCATTATGATCTGATTCTCGTGGGGGCTGGACTCGCGAATGGCCTTATCGCCCTGCGTCTTCAGCAGC
AGCAACCTGATATGCGTATTTTGCTTATCGACGCCGCACCCCAGGCGGGCGGGAATCATACGTGGTCATTTCACCA
CGATGATTTGACTGAGAGCCAACATCGTTGGATAGCTCCGCTGGTGGTTCATCACTGGCCCGACTATCAGGTACGC
TTTCCCACACGCCGTCGTAAGCTGAACAGCGGCTACTTTTGTATTACTTCTCAGCGTTTCGCTGAGGTTTTACAGC

GACAGTTTGGCCCGCACTTGTGGATGGATACCGCGGTCGCAGAGGTTAATGCGGAATCTGTTCGGTTGAAAAAGGG
TCAGGTTATCGGTGCCCGCGCGGTGATTGACGGGCGGGGTTATGCGGCAAATTCAGCACTGAGCGTGGGCTTCCAG
GCGTTTATTGGCCAGGAATGGCGATTGAGCCACCCGCATGGTTTATCGTCTCCCATTATCATGGATGCCACGGTCG
ATCAGCAAAATGGTTATCGCTTCGTGTACAGCCTGCCGCTCTCGCCGACCAGATTGTTAATTGAAGACACGCACTA
TATTGATAATGCGACATTAGATCCTGAATGCGCGCGGCAAAATATTTGCGACTATGCCGCGCAACAGGGTTGGCAG
CTTCAGACACTGCTGCGAGAAGAACAGGGCGCCTTACCCATTACTCTGTCGGGCAATGCCGACGCATTCTGGCAGC
AGCGCCCCCTGGCCTGTAGTGGATTACGTGCCGGTCTGTTCCATCCTACCACCGGCTATTCACTGCCGCTGGCGGT
TGCCGTGGCCGACCGCCTGAGTGCACTTGATGTCTTTACGTCGGCCTCAATTCACCATGCCATTACGCATTTTGCC
CGCGAGCGCTGGCAGCAGCAGGGCTTTTTCCGCATGCTGAATCGCATGCTGTTTTTAGCCGGACCCGCCGATTCAC
GCTGGCGGGTTATGCAGCGTTTTTATGGTTTACCTGAAGATTTAATTGCCCGTTTTTATGCGGGAAAACTCACGCT
GACCGATCGGCTACGTATTCTGAGCGGCAAGCCGCCTGTTCCGGTATTAGCAGCATTGCAAGCCATTATGACGACT
CATCGTTAATAA

Supplementary Table 6: Primers used in this study

Promoter	Assembly of dual-input promoters	
pLacZnu1	pLacZnu1.prefix.F	GAATTCGCGGCCGCTTCTAGAAATTGTGAGCGGATAACAATTGACA TTGTGAGCGGATAACAAGATACTGAGCACAGAAGT
	pLacZnu1.suffix.R	CTGCAGCGGCCGCTACTAGTAAAATGTTATAATATCACACTTCTGT GCTCAGTATCTTGTTA
pLacZnu2A	ZurO.LacO.R	TTGTTATCCGCTCACAATTAAATGTTATAATATCACACTTCTGTGC TCAGTATC
	suffix.LacOID.F	TTTAATTGTGAGCGGATAACAATACTAGTAGCGGCCGCT
pLacZnu2B	ZurOLacO.R	AATTGTGAGCGCTCACAATTCAGTCTTGTTATCCGCTCA
	suffix.LacO2.F	AATTGTGAGCGCTCACAATTTACTAGTAGCGGCCGCTG
pBadZnu1	pBad.ZurO.R	AAATGTTATAATATCACACTTCTCATATTCATCATCAGGTAGGATCCGC TAATCTTA
	ZurOsuf.F	GAAGTGTGATATTATAACATTTTACTAGTAGCG
pT7Znu1	pT7.LacOZurO.R	AAATGTTATAATATCACACTTCTTGTTATCCGCTCACAATTCCTAT AGTGAGTCGTATTATCTAGAAGC
	ZurOsuf.F	GAAGTGTGATATTATAACATTTTACTAGTAGCG
pT7Znu2A	ZurO.R	AATTGTGAGCGCTCACAATTAAATGTTATAATATCACACTTCTTGTTAT CCGCTC
	suffix.LacO2.F	AATTGTGAGCGCTCACAATTTACTAGTAGCGGCCGCTG
pT7Znu2B	ZurO.F	GAAGTGTGATATTATAACATTTAATTGTGAGCG
	ZurOLacO.R	AAATGTTATAATATCACACTTCTTGTTATCCGC

Modulation of Zur expression levels	
ssRAForward.Prefix	GAATTCGCGGCCGCTTCTAGAGGCTGCTAACGACGAAAACTAC
M50.spe1.R	GCTACTAGTATTAAGCAGCCAGAGCGTAGTTTTCGTCGTTAGCAGC
M51.spe1.R	GCTACTAGTATTAAGAAGCGTCAGCGTAGTTGTAGTTTTCGTCGTTAGCAGC
M52.spe1.R	GCTACTAGTATTAAGAAGCGTCAGCGTAGTTTTCGTCGTTAGCAGC
Zur.ssra.R	GTAGTTTTCGTCGTTAGCAGCACGCGGTTTCTTTTTCACCTG
ssrAgeneral.ZurOverlap.F	GGTGAAAAAGAAACCGCGTGCTGCTAACGACGAAAACTAC
Zur.r100.F	TAATTAACCCCAAGCCAAGCGGCGTATGGAAAAGACCACAACGCAG
pZntA.r100.R	CGCTTGGCTTGGGGTTAATTAGGTCCCATTAACCGAAGGATACACTCTGGA
Zur.r300.F	GTAATATTTCGAGAATTTGAGATCAATGGAAAAGACCACAACGCAG
pZntA.r300.R	TCAAATTCTCGAAATATTACATTAACCGAAGGATACACTCTGGA
Assembly of library of Zur mutants	
zurH89.NNK.F	AGTTATGTGCTCTGTNNKCTGTTCGATC
zurH89.R	ACAGAGCACATAACTGTTGGTG
zurL90.NNK.F	TTATGTGCTCTGTCATNNKTTCGATCAGC
zurL90.R	ATGACAGAGCACATAACTGTTGGTG
zurQ93.NNK.F	TCTGTCATCTGTTCGATNNKCCCACC
zurQ93.R	ATCGAACAGATGACAGAGCAC

zurP94.NNK.F	CATCTGTTCGATCAGNNKACCCATACG
zurP94.R	CTGATCGAACAGATGACAGAGC
zurI120.NNK.F	GAAGGCGTGGAAGACNNKATGCAT
zurI120.R	GTCTTCCACGCCTTCTGCAC
zurL124.NNK.F	AGACATTATGCATACGNNKGCGGC
zurL124.R	CGTATGCATAATGTCTTCCACGC
zurL142.NNK.F	ATTGAAGCACATGGGNNKTGTGCGG
zurL142.R	CCCATGTGCTTCAATCACATTATG
ZurC103S.F	CATGTTTATTTCAGATCGCTGCGG
ZurC103S.R	CCGCAGCGATCTGAAATAAACATG
Untargeted mutagenesis of Zur	
Zur.F	ATGGAAAAGACCACAACGCAG
Zur.R	TTAACGCGGTTTCTTTTTCACCTG
117.31Zur.R	GTTGTGGTCTTTTCCATCTAGTAGGTTTCCTGTGTGAGCTAGCACAATCCCTAGGA
ZurEnd.F	CAGGTGAAAAAGAAACCGCGTTAA
Creation and screening of Δ*zur* strain	
p1.ZurOvlp.F	AACATGGTAAAGTAAGGACATTCTTAACCCCCACTTTGAGGTGCCCGATGGTGTAGG CTGGAGCTGCTT
p2.ZurOvlp.R	GCCCGACGTGTACAAGGATGTACGCCCTCTTAACGCGGTTTCTTTTTCACTGGGAAT TAGCCATGGTCC
ZurUS.299.F	GTCGTACCGTGTCCTCCTTA
ZurDS.189.R	GGGCAAACTGACCGATGATG
Assembly anchor points and screening plasmids	
ZurMid.F	GTGCTCTGTCATCTGTTCGA
ZurMid.R	TCGAACAGATGACAGAGCAC
crtEmid.F	CTTGACGATATGCCCTGCAT
crtEmid.R	ATGCAGGGCATATCGTCAAG
crtImid.F	GTTACTATGTGTTGGCGCCG
crtImid.R	CGGCGCCAACACATAGTAAC
lacImid.F	GACGATACCGAAGACAGCTC
lacImid.R	GAGCTGTCTTCGGTATCGTC
vioDmid.F	CCAGCTGTTCGATCAGATGA
vioDmid.R	TCATCTGATCGAACAGCTGG
vioAmid.F	CTCAGTTGCACCCGCATTT
vioAmid.R	AAATGCGGGTGCAACTGAG
VR	ATTACCGCCTTTGAGTGAGC
VF2	TGCCACCTGACGTCTAAGAA

VRinv	GCTCACTCAAAGGCGGTAAT
VF2 inv	TTCTTAGACGTCAGGTGGCA

References

1 Salis, H. M., Mirsky, E. A. & Voigt, C. A. Automated design of synthetic ribosome binding sites to control protein expression. *Nature Biotechnology* **27**, 946-U112, doi:10.1038/nbt.1568 (2009).

2 Gilston, B. A. *et al.* Structural and mechanistic basis of zinc regulation across the *E. coli* Zur regulon. *PLoS biology* **12**, e1001987, doi:10.1371/journal.pbio.1001987 (2014).

3 Schrodinger, L. The PyMOL Molecular Graphics System, Version~1.8. (2015).

4 Watstein, D. M., McNerney, M. P. & Styczynski, M. P. Precise metabolic engineering of carotenoid biosynthesis in *Escherichia coli* towards a low-cost biosensor. *Metabolic engineering* **31**, 171-180, doi:10.1016/j.ymben.2015.06.007 (2015).
