# Supplementary material for: Dynamic and tunable metabolite control for robust minimal-equipment assessment of serum zinc
Source: Nat Commun. 2019 Dec 4;10:5514. doi: 10.1038/s41467-019-13454-1 (PMC6892929; doi:10.1038/s41467-019-13454-1)
Supplement: Supplementary file 3 — Reporting Summary [file 41467_2019_13454_MOESM3_ESM.pdf]

nature research

Corresponding author(s): Mark P. Styczynski
Last updated by author(s): 09/16/2019

Reporting Summary

Nature Research wishes to improve the reproducibility of the work that we publish. This form provides structure for consistency and transparency in reporting. For further information on Nature Research policies, see Authors & Referees and the Editorial Policy Checklist.

Statistics

For all statistical analyses, confirm that the following items are present in the figure legend, table legend, main text, or Methods section.

n/a	Confirmed	
☐	☒	The exact sample size (*n*) for each experimental group/condition, given as a discrete number and unit of measurement
☐	☒	A statement on whether measurements were taken from distinct samples or whether the same sample was measured repeatedly
☒	☐	The statistical test(s) used AND whether they are one- or two-sided<br>*Only common tests should be described solely by name; describe more complex techniques in the Methods section.*
☒	☐	A description of all covariates tested
☒	☐	A description of any assumptions or corrections, such as tests of normality and adjustment for multiple comparisons
☒	☐	A full description of the statistical parameters including central tendency (e.g. means) or other basic estimates (e.g. regression coefficient) AND variation (e.g. standard deviation) or associated estimates of uncertainty (e.g. confidence intervals)
☒	☐	For null hypothesis testing, the test statistic (e.g. F, t, r) with confidence intervals, effect sizes, degrees of freedom and P value noted<br>*Give P values as exact values whenever suitable.*
☒	☐	For Bayesian analysis, information on the choice of priors and Markov chain Monte Carlo settings
☒	☐	For hierarchical and complex designs, identification of the appropriate level for tests and full reporting of outcomes
☒	☐	Estimates of effect sizes (e.g. Cohen's d, Pearson's r), indicating how they were calculated

Our web collection on statistics for biologists contains articles on many of the points above.

Software and code

Policy information about availability of computer code

Data collection	An Android app that was developed for this manuscript was used to analyze images of cell pellets
Data analysis	The code used for analysis is freely available on GitHub (https://github.com/gtStyLab/zinQapp)

For manuscripts utilizing custom algorithms or software that are central to the research but not yet described in published literature, software must be made available to editors/reviewers. We strongly encourage code deposition in a community repository (e.g. GitHub). See the Nature Research guidelines for submitting code & software for further information.

Data

Policy information about availability of data

All manuscripts must include a data availability statement. This statement should provide the following information, where applicable:

- Accession codes, unique identifiers, or web links for publicly available datasets
- A list of figures that have associated raw data
- A description of any restrictions on data availability

The raw data used in this study is provided as in a supplementary spreadsheet.

Field-specific reporting

Please select the one below that is the best fit for your research. If you are not sure, read the appropriate sections before making your selection.

☒ Life sciences ☐ Behavioural & social sciences ☐ Ecological, evolutionary & environmental sciences

For a reference copy of the document with all sections, see nature.com/documents/nr-reporting-summary-flat.pdf

Life sciences study design

All studies must disclose on these points even when the disclosure is negative.

Sample size	All reported measurements are the average of biological triplicates, as is standard for fluorescent and colorimetric analyses to give an indication of the variability in the data. A detailed power calculation was not necessary because we were not performing statistical tests.
Data exclusions	No data were excluded from the analyses.
Replication	Pigmentation was qualitatively assessed in two different media batches on two different days to ensure reproducibility of coloration.
Randomization	Randomization was not relevant to this study, as samples were not allocated into groups and instead all processed simultaneously.
Blinding	Blinding was not relevant to this study because samples were not allocated into groups.

Reporting for specific materials, systems and methods

We require information from authors about some types of materials, experimental systems and methods used in many studies. Here, indicate whether each material, system or method listed is relevant to your study. If you are not sure if a list item applies to your research, read the appropriate section before selecting a response.

Materials & experimental systems

n/a	Involved in the study	
☒	☐	Antibodies
☒	☐	Eukaryotic cell lines
☒	☐	Palaeontology
☒	☐	Animals and other organisms
☐	☒	Human research participants
☒	☐	Clinical data

Methods

n/a	Involved in the study	
☒	☐	ChIP-seq
☒	☐	Flow cytometry
☒	☐	MRI-based neuroimaging

Human research participants

Policy information about studies involving human research participants

Population characteristics	Blood was taken from healthy volunteers of both genders. The following populations were excluded from this study due to Georgia Tech IRB policy: pregnant volunteers, children (under the age of 18), those with history of immunodeficiency or HIV infection, those with history of allergy to latex, those who had donated blood during the immediate past 8 week period, those with suspected anemia.
Recruitment	Recruitment was done by word of mouth in laboratories at Georgia Tech.
Ethics oversight	The study was approved by Georgia Tech's Institutional Review Board (Protocol H17489)

Note that full information on the approval of the study protocol must also be provided in the manuscript.
